# Analysis of the Impact of Fault Mechanism Radiation Patterns on Macroseismic Fields in the Epicentral Area of 1998 and 2004 Krn Mountains Earthquakes (NW Slovenia)

**DOI:** 10.1155/2014/206843

**Published:** 2014-03-19

**Authors:** Andrej Gosar

**Affiliations:** ^1^Faculty of Natural Sciences and Engineering, University of Ljubljana, Aškerčeva 12, 1000 Ljubljana, Slovenia; ^2^Slovenian Environment Agency, Seismology and Geology Office, Dunajska 47, 1000 Ljubljana, Slovenia

## Abstract

Two moderate magnitude (Mw = 5.6 and 5.2) earthquakes in Krn Mountains occurred in 1998 and 2004 which had maximum intensity VII-VIII and VI-VII EMS-98, respectively. Comparison of both macroseismic fields showed unexpected differences in the epicentral area which cannot be explained by site effects. Considerably, different distribution of the highest intensities can be noticed with respect to the strike of the seismogenic fault and in some localities even higher intensities have been estimated for the smaller earthquake. Although hypocentres of both earthquakes were only 2 km apart and were located on the same seismogenic Ravne fault, their focal mechanisms showed a slight difference: almost pure dextral strike-slip for the first event and a strike-slip with small reverse component on a steep fault plane for the second one. Seismotectonically the difference is explained as an active growth of the Ravne fault at its NW end. The radiation patterns of both events were studied to explain their possible impact on the observed variations in macroseismic fields and damage distribution. Radiation amplitude lobes were computed for three orthogonal directions: radial P, SV, and SH. The highest intensities of both earthquakes were systematically observed in directions of four (1998) or two (2004) large amplitude lobes in SH component (which corresponds mainly to Love waves), which have significantly different orientation for both events. On the other hand, radial P direction, which is almost purely symmetrical for the strike-slip mechanism of 1998 event, showed for the 2004 event that its small reverse component of movement has resulted in a very pronounced amplitude lobe in SW direction where two settlements are located which expressed higher intensities in the case of the 2004 event with respect to the 1998 one. Although both macroseismic fields are very complex due to influences of multiple earthquakes, retrofitting activity after 1998, site effects, and sparse distribution of settlements, unusual differences in observed intensities can be explained with different radiation patterns.

## 1. Introduction

**Figure 1:**
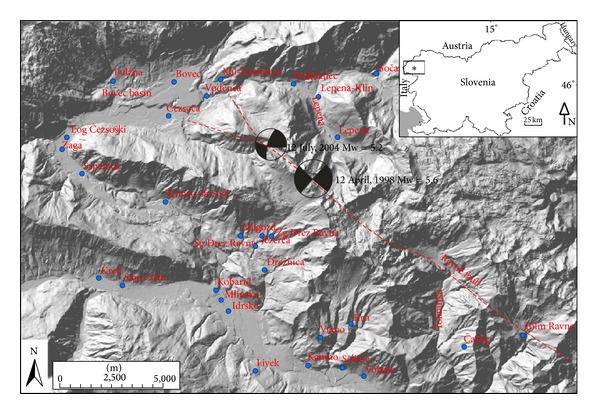
Epicentral area of 1998 and 2004 Krn Mountains earthquakes with both focal mechanisms, the trace of seismogenic Ravne fault and settlements, for which intensity data are available.

**Figure 2:**
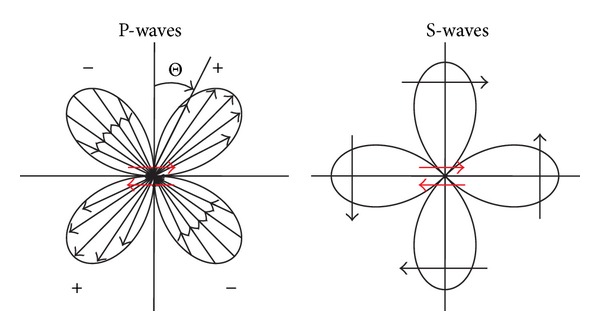
Diagrams of radiation patterns of the radial displacement component of P-waves (left) and of the transverse displacement component of S-waves (right) (after [12] and [13]).

In 1998 and 2004 two moderate magnitude (Mw = 5.6 and 5.2) earthquakes (Figure 1) occurred in Krn Mountains (NW Slovenia) in an area where weak seismicity was characteristic during the last century, although the wider area (Upper Soča valley) is recognized by relatively high seismic hazard due to its proximity to very active adjacent Friuli area (NE Italy). Both earthquakes occurred in sparsely populated area characterized by high mountains. The maximum intensity of 1998 event was VII-VIII EMS-98 [1] and of the 2004 event was VI-VII EMS-98 [2]. Relatively strong variations in damage to buildings and assessed intensities were observed in the area, especially in the Bovec basin at similar epicentral distances. They could not be explained by different building vulnerability, since the building typology is similar throughout the area. Local geological conditions (site effects) proved to be the most important factor of observed intensity variations in a single earthquake. They were intensively studied in the last decade especially with microtremor methods and focused on soil-structure resonance phenomena [3–6]. Another reason for the observed variations in damage distribution at similar epicentral distances could be the impact of the fault mechanism radiation pattern of seismic energy from the source. This possible effect attracted more attention after the second earthquake in 2004 [7], which occurred on the same fault only 2 km apart from the 1998 event, because the distribution of observed intensities in the epicentral area was considerably different than in the 1998 event. Although both earthquakes occurred very close, their focal mechanisms were slightly different, almost pure dextral strike-slip for the 1998 event, whereas the 2004 event has also a small reverse component besides its predominantly strike-slip character (Figure 1). These observations motivated a detailed study of radiation patterns of both Krn Mountain earthquakes and of their possible impact on observed macroseismic fields (damage distribution) in epicentral area which is presented in this paper. Each earthquake shear rupture shows a typical radiation pattern, which is different for body (P and S) waves and surface (Rayleigh and Love) waves. These patterns are characterized by amplitude lobes that present azimuth-dependent radiation of energy (Figure 2). Detailed analysis of radiation patterns can reveal a relation between the radiated energy for different wave types and observed macroseismic fields. Source effects on the distribution of macroseismic intensity were recently studied for different earthquakes (e.g. [8, 9]).

## 2. Seismotectonic Characteristics

The Upper Soča valley is located in one of the three areas of the highest seismic hazard in Slovenia. This is mainly due to its proximity to the seismically very active area of Friuli located 30–40 km to the W in NE Italy. In this area, the Mw = 6.4 Friuli earthquake occurred in May 1976. The seismic sequence that followed consists of six aftershocks of Mw between 5.0 and 6.0 in the same year [10]. For adjacent NW Slovenia, relatively weak rate of seismicity was characteristic before April 1998 when Mw = 5.6 earthquake occurred in Krn Mountains. It was followed by a Mw = 5.2 event in July 2004 in the same epicentral area. Both earthquakes occurred on the NW-SE trending near-vertical dextral strike-slip Ravne fault [11] at 7.6 and 11 km depth, respectively. Maximum intensity of 1998 earthquake was VII-VIII EMS-98 [1] and of the 2004 event was VI-VII EMS-98 [2].

NW Slovenia and Friuli region are located at the kinematic transition between E-W striking trust faults of the Alpine system (Friuli earthquakes) and NW-SE striking strike-slip faults of the Dinarides system (Krn Mountains earthquakes). The strongest earthquake ever recorded in the Alps-Dinarides junction area was the 1511 western Slovenia earthquake (*M* = 6.8); the exact location and mechanism of this event are still debated [14, 15]. According to the seismic hazard map of Slovenia for a 475-year return period [16], a design ground acceleration value for a rock site in majority of the Upper Soča valley is 0.225 g, but in the most western part close to the border with Italy, it is 0.250 g.

The seismogenic Ravne fault (Figure 1) is an actively propagating NW-SE trending dextral strike-slip fault. Strike-slip displacements on steep fault planes are responsible for the recent seismic activity that is confined to shallow crustal levels [11, 17]. The fault is growing by interaction of individual right stepping fault segments and breaching of local transtensional step-over zones. The fault geometry is controlled by the original geometry of the NW-SE trending thrust zone, modified by successive faulting within the fault zone. In the recent N-directed maximum horizontal stress regime, the segmented fault is lengthening by an active growth at the fault's NW end. The spatial distribution of earthquake clusters shows that activity on strike-slip segments and thrust faults is contemporaneous [11].

Using the strong motion inversion [18] revealed that the rupture of the 1998 event was confined between 3 and 9 km depth and propagated bilaterally within the two structural barriers (Bovec basin and Tolminka spring basin) in 12 km long segment of the Ravne fault (Figure 1).

Fault plane solutions were determined from the waveforms recorded with broad-band seismographs and accelerographs in Slovenia, NE Italy, and Austria using the polarities and amplitudes of the first arrival of longitudinal and transversal waves (Table 1). For the 1998 main shock, the fault plane solution shows almost pure dextral strike-slip on NW-SE trending fault (Figure 1), but solutions of 21 stronger (*M*_*L*_ ≥ 2.5) aftershocks are different [1]. The aftershocks have mainly reverse component; the direction of the plane is WNW-ESE and both possible solutions have similar dip. The major principal stress component is almost horizontal in N-S direction. Most of aftershock hypocentres were shallower than the main shock [1]. For the 2004 main shock, the fault plane solution shows predominantly dextral strike-slip kinematics with a small component of reverse movement on a steep, towards SW dipping plane (Figure 1). Fault plane solutions of eight stronger (*M*_*L*_ ≥ 2.4) aftershocks were also derived and analysed [19]. First aftershock cluster occurred NW of the main shock and showed similar kinematics as the main event, but the fault planes dip to the NE. Fault plane solutions of the second aftershock cluster located in the area of Čezsoča village (Figure 1) show, compared to the others, more moderate to shallow dipping fault planes oriented in E-W direction. These events show almost purely reverse movements on shallowly dipping fault planes to oblique reverse-dextral strike-slip faulting on moderate to steep E-W trending faults [19]. Distribution of aftershocks and their fault plane solutions supports the idea that activity on strike-slip segments and thrust faults is contemporaneous and that segmented Ravne fault is lengthening by an active growth at its NW end [11].

The epicentres of the 1998 and 2004 events are located in an area of poor surface fault exposure. Additionally, the main shocks of both events are shifted to the NW relative to the overall spatial distribution of aftershocks and focal mechanisms of aftershocks suggest reactivation of older structures in the strike-slip tectonic regime [11]. In Figure 1, two branches of presumable surface trace of the fault are shown, which terminate close to the Bovec basin, which represents a structural barrier.

## 3. Macroseismic Fields of 1998 and 2004 Earthquakes

**Figure 3:**
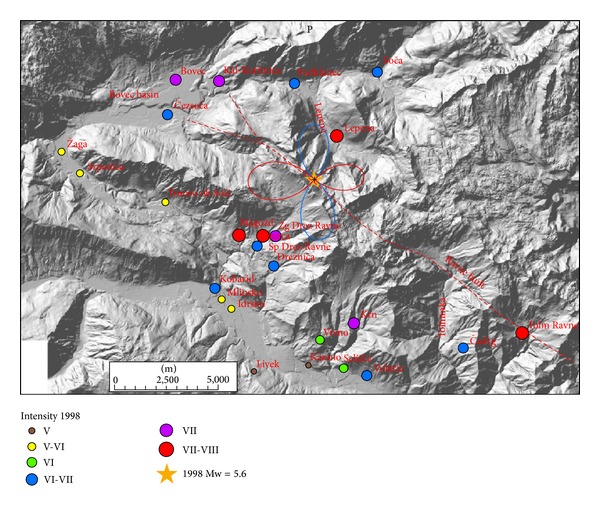
Diagram of radial P component for 1998 earthquake and intensity data in the epicentral area. Positive lobes are shown in red and negative lobes in blue colour.

Macroseismic data were collected by field investigations, by macroseismic questionnaires, and by contributions of damage inspection commissions [1]. The data on damage to buildings and other earthquake effects were analysed according to the definitions and guidelines of European Macroseismic Scale 1998 (EMS-98), which is predominantly used in Europe in the last two decades [20]. Macroseismic data of more than 2000 localities were evaluated in Slovenia and relevant data were obtained also from neighbouring countries (Italy, Austria). Since the epicentres were located in uninhabited high mountain area, the data from the epicentral area is relatively sparse, limited to some settlements in the nearby valleys (Figure 1). The macroseismic fields of both earthquakes were thus very unevenly sampled. Extensive effects on natural environment (more than 78 rockfalls occurred in mountains) were studied in addition using Environmental Seismic Intensity scale [21]. For the 1998 earthquake, the maximum intensity VII-VIII EMS-98 was observed in Lepena, Magozd, Spodnje Drežniške Ravne, and Tolminske Ravne [1]. In the Bovec basin, intensities were slightly lower, VII in Bovec and Kal-Koritnica and VI-VII in Čezsoča (Table 2, Figure 3)

The most common type of building in the area is masonry. Older buildings are made of simple and massive stone, with wooden floors. Modern buildings are reinforced masonry with reinforced concrete floors. Some industrial and commercial buildings are made of reinforced concrete frames or walls. There are no high-rise buildings in this area. After the 1998 earthquake, more than 3000 houses were examined by engineers and their damage was described in detail. The typical damage of the older stone buildings with wooden floors was partial collapse of walls or corners. Many houses had damage on roofs and chimneys and deep and extensive cracks in walls were often seen. Some newer masonry buildings were also damaged and their damage was in many cases enhanced by unfavourable soil conditions [1].

Intensive retrofitting activities took place after the 1998 earthquake but were not completely finished before the 2004 earthquake. This fact partly influenced the assessment of the 2004 event intensities. Some of the retrofitted houses were damaged in the second earthquake as well. The maximum intensity of VI-VII EMS-98 was observed in Bovec, Čezsoča, and Vodenca (Table 2, Figure 6), all located in the Bovec basin.

Comparison of 1998 and 2004 intensities in settlements of the Upper Soča valley (Table 2, Figures 3 and 6) shows some unexpected features of macroseismic fields. Although the 1998 earthquake was considerably larger in magnitude and has also one degree larger maximum intensity, this difference is not reflected uniformly in all settlements. For the 1998 event, maximum intensity VII-VIII was observed SW (Magozd, Sp. Drežniške Ravne) and NE (Lepena) of the epicentre, both at the epicentral distance < 2.5 km, and in Tolminske Ravne, 7 km to the SE (Figure 3). The last settlement is located on a glacial moraine, on which moderate site effects are expected, that very likely enhanced the intensity. On the other hand, for the 2004 event maximum intensity VI-VII was observed in the Bovec basin (Bovec, Čezsoča, and Vodenca), whereas intensities in Magozd and Lepena are for 1.5 degrees lower and in Sp. Drežniške Ravne for 2 degrees lower in comparison to the 1998 event (Figure 6). For the 2004 earthquake, not enough data was collected in Tolminske Ravne to enable its intensity estimate. The difference in intensity of both events in Bovec and Kal-Koritnica is 1 degree, but in Čezsoča the same intensity (VI-VII) was assessed for both earthquakes. For two settlements (Srpenica and Trnovo ob Soči), the assessed intensity for the 2004 event was even larger by 0.5 degree.

## 4. Fault Mechanism Radiation Patterns

### 4.1. Methodology

In case of a tectonic earthquake, the two adjacent rock formations on both sides of the fault move relatively to each other in the opposite direction. The radiation pattern of energy emitted as seismic waves can be modelled using the double force couple using the approach that does not involve a fault discontinuity. The double couple is mathematically described in 3 dimensions by a symmetrical tensor with 9 components known as moment tensor.

Earthquake shear ruptures show a typical radiation pattern, which is different for body (P and S) waves and surface (Rayleigh and Love) waves. These patterns are characterized by amplitude lobes, that is by azimuth-dependent radiation of amplitudes, respectively energy (Figure 2). In the case of a pure shear rupture in a homogeneous isotropic medium, P-waves have the smallest (idealized zero) amplitudes in the direction of the fault rupture and the largest amplitudes at an angle 45° off the plane, whereas S-waves have the largest amplitudes in the direction of the shear rupture and perpendicular to it (Figure 2) [13].

Radiation patterns are analysed in three orthogonal directions: radial P, vertical SV, and horizontal SH. The same three directions are used to analyse surface waves, which have normally the largest amplitudes. Due to their elliptical and retrograde motion, Rayleigh waves are analysed in P and SV directions, whereas Love waves, which are similar to horizontally polarised S-waves, are analysed in SH direction. It is well known that S-waves and Love waves normally constitute the most powerful part of the ground motion and thus cause the highest damage to buildings. Not only do these phases carry the strongest energy to near and regional distances, but also the frequencies contained span the band from 0.1 to 10.0 Hz, which is of the main engineering interests [22]. The observed P, SV, and SH lobe pattern depends on the fault's strike, dip, and rake and thus on the source mechanism type of the acting fault plane, as well as on the incidence angle of the respective seismic rays at the target location, which depends on its epicentral distance and the source depth.

Six different scalar products are needed to describe radiation pattern. In dimensionless form, the radiation patterns in three orthogonal directions are given by [12]

(1) FP=cos⁡λsinδ sin2⁡isin⁡2(ϕ−ϕS)−cos⁡λcos⁡δsin⁡2icos⁡(ϕ−ϕS)+sinλsin⁡2δ(cos⁡2⁡i−sin2⁡i sin2⁡(ϕ−ϕS))+sinλcos⁡2δsin2isin(ϕ−ϕS)FSV=sinλcos⁡2δcos⁡2isin(ϕ−ϕS)−cos⁡λcos⁡δcos⁡2icos⁡(ϕ−ϕS)+12cos⁡λsinδsin2isin2(ϕ−ϕS)+12sinλsin2δsin2i(1+sin2(ϕ−ϕS))FSH=cos⁡λcos⁡δcos⁡isin(ϕ−ϕS)−cos⁡λcos⁡δsinicos⁡2(ϕ−ϕS)+sinλcos⁡2δcos⁡icos⁡(ϕ−ϕS)+12sinλsin2δsinisin2(ϕ−ϕS),

where *ϕ*_*S*_ is strike, *δ* is dip, *λ* is rake, *i* is take-off angle, and *ϕ* is source receiver azimuth.

Each radiation pattern is composed of positive and negative amplitude lobes plotted usually in different colours against azimuth, using appropriate software for plotting 2D functions and presented separately for each of the three orthogonal directions (e.g., Figure 3). In addition three-dimensional presentation of amplitude lobes can be used as an illustration which can be rotated and viewed from different angles but is not so suitable for quantitative interpretation.

### 4.2. Radiation Patterns of 1998 and 2004 Earthquakes

Conventional presentation of earthquake fault plane solutions as “seismological beach ball plots” (Figure 1) normally used in seismotectonic studies is very powerful for seismotectonic interpretations but not suitable to understand all differences in radiation patterns related to relatively small variations in strike, dip, and rake. The difference in fault plane solutions between 1998 and 2004 earthquakes from focal mechanisms shown in Figure 1 seems to be relatively small. The main difference is that the 1998 event shows almost pure dextral strike-slip motion on almost vertical (dip is 86°) NW-SE trending fault, whereas the 2004 event shows predominantly dextral strike-slip motion with a small component of reverse movement on a steep, towards SW dipping plane (dip is 72°). The main question of our study was if this difference has sufficient influence on the radiation pattern that it can cause the observed variations in macroseismic fields of both earthquakes.

**Figure 4:**
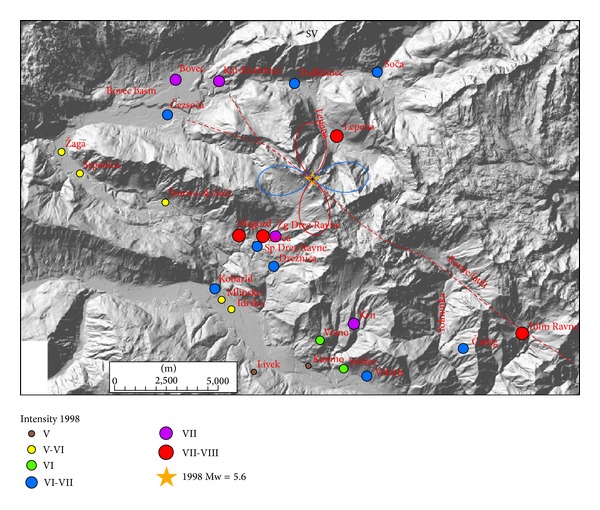
Diagram of SV component for 1998 earthquake and intensity data in the epicentral area. Positive lobes are shown in red and negative lobes in blue colour.

**Figure 5:**
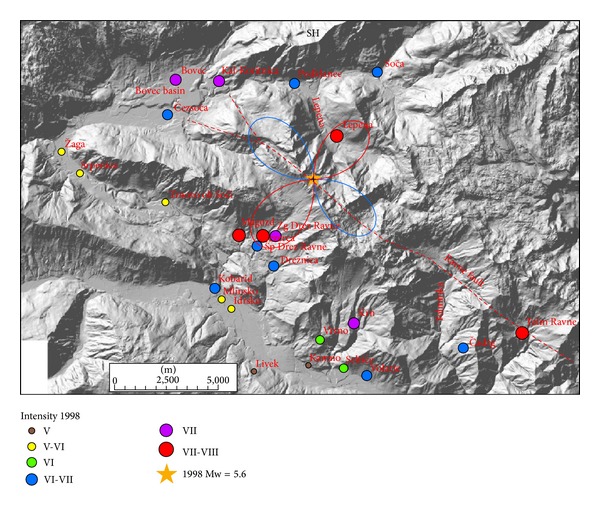
Diagram of SH component for 1998 earthquake and intensity data in the epicentral area. Positive lobes are shown in red and negative lobes in blue colour.

**Figure 6:**
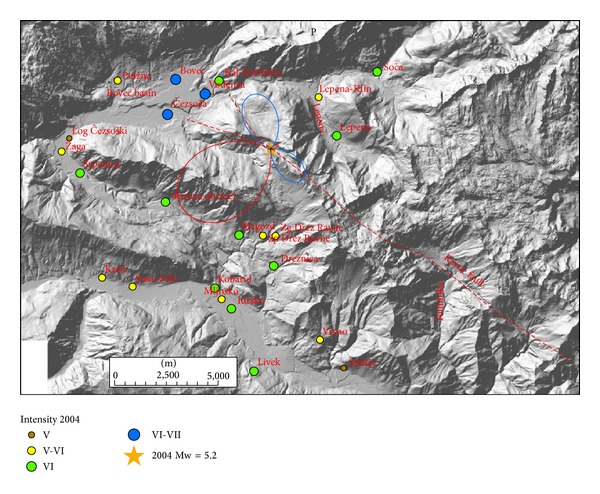
Diagram of radial P component for 2004 earthquake and intensity data in the epicentral area. Positive lobes are shown in red and negative lobes in blue colour.

**Figure 7:**
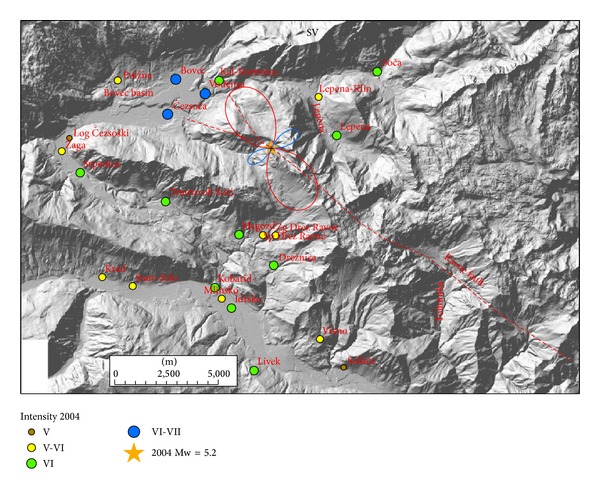
Diagram of SV component for 2004 earthquake and intensity data in the epicentral area. Positive lobes are shown in red and negative lobes in blue colour.

**Figure 8:**
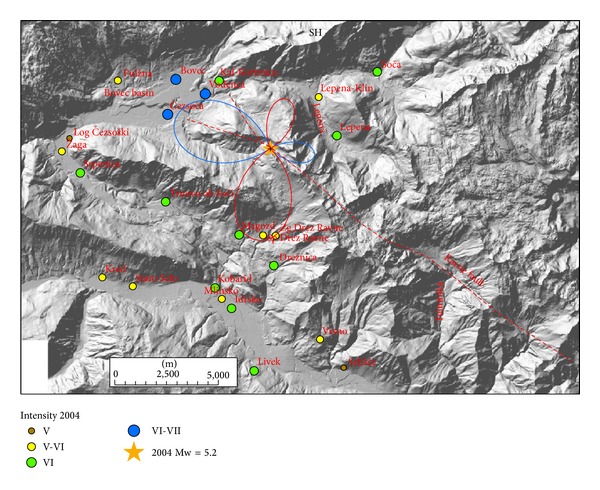
Diagram of SH component for 2004 earthquake and intensity data in the epicentral area. Positive lobes are shown in red and negative lobes in blue colour.

Radiation patterns were computed using (1) and presented on separate figures for three orthogonal directions (radial P, vertical SV, and horizontal SH) together with macroseismic data of each event. Positive amplitude lobes are presented in red and negative lobes in blue colour. Radiation patterns of the 1998 earthquake are presented in Figures 3–5 and of the 2004 earthquake in Figures 6–8. For all plots, the same scale is used.

Radiation patterns of the 1998 event reflect almost pure dextral strike-slip motion on almost vertical fault plane. Radiation patterns are thus symmetrical and very similar to idealized case shown in Figure 2. Radial P (Figure 3) and SV (Figure 4) components have the smallest amplitudes in the direction of the fault rupture and the largest amplitudes at an angle 45° off the plane. The signature of the positive and negative lobes in SV direction is reversed with respect to P direction. The difference in the shape of the lobes is very small; only the western lobe in P direction is slightly larger than the eastern one. On the other hand, SH direction (Figure 5) shows the largest amplitudes in the direction of the shear rupture and perpendicular to it. The amplitudes of all four lobes are larger in comparison to P and SV radiation patterns. SH direction corresponds mainly to Love waves, which normally represents the most destructive part of the ground shaking. Figure 5 obviously shows that the settlements with the highest observed intensity (VII-VIII EMS) for 1998 earthquake lie in the direction of all four SH lobes. Magozd and Sp. Drežniške Ravne are in the direction of positive SW lobe; Lepena is in the direction of opposite NE lobe; Tolminske Ravne with VII-VII intensity is in the direction of negative SE lobe; and Bovec and Kal-Koritnica with VII intensity are in the direction of opposite NW lobe. Third settlement in the Bovec basin-Čezsoča, although located closer to the epicentre then Bovec, has lower intensity (VI-VII). This can be explained by the fact that it lies already out of the direction of the lobe. The same is valid for Podklanec (VI-VII), north of the epicentre, located in the direction of almost the smallest SH amplitude.

Radiation patterns of the 2004 event reflect a clear deviation of fault mechanism from pure dextral strike-slip, because it has also a reverse component on a steep dipping plane. Radiation patterns are thus highly asymmetric. Radial P component (Figure 6) shows the largest positive lobe in SW direction and a very small lobe in opposite NE direction. In SW direction settlements, Srpenica and Trnovo ob Soči express intensity VI, which is higher than intensity V-VI related to the 1998 event. On the other hand, for Lepena site located in NE direction, the intensity in 2004 was much lower (VI) in comparison to the 1998 event (VII-VIII). Such difference in macroseismic field can be thus related to the highly asymmetric shape of radial P radiation pattern. On the other hand, there is no clear correlation between damage distribution and SV radiation pattern (Figure 7), which is rather symmetrical and has large positive lobes in the direction of the strike and small negative lobes in the cross-direction. SH radiation pattern (Figure 8) shows the largest negative amplitude lobe in direction of the Bovec basin (WNW) but oriented for approximately 30° more towards the west with respect to the 1998 radiation lobe for the same component. This can explain why the highest intensity (VI-VII) of the 2004 event was observed not only in Bovec and Vodenca but also in Čezsoča, located at the southern margin of the Bovec basin. On the other hand, Kal-Koritnica, which expresses lower intensity (VI) for the 2004 event, is located already out of the direction of the lobe. The second large positive lobe is oriented towards SSW where settlements Magozd, Drežnice, and Drežniške Ravne with observed intensities VI or V-VI are located. Also for this event, we can conclude that the largest ground motion and thus damage are related to SH direction (Love waves) with the largest lobe in direction of the Bovec basin. In addition, very interesting is the increase of intensity of the 2004 event in SW-located settlements (Srpenica and Trnovo ob Soči) which can be related to the very large lobe in radial *P* direction (Figure 6) as described above. This is the most prominent asymmetric feature of the 2004 event radiation pattern.

## 5. Discussion of Results and Conclusions

Analyses of radiation patterns of 1998 and 2004 earthquakes have shown that they can contribute to the understanding of the unusual differences observed in macroseismic fields of both events. Most interesting is a clearly different distribution of the largest observed intensities for both earthquakes and two localities which showed higher intensities for the smaller 2004 earthquake with respect to the larger 1998 event. Since the epicentres of both events are located very close, these differences cannot be related to eventual 2D or 3D site effects in the sedimentary basins. On the other hand, focal mechanisms show a difference, which seems at a first glance small but has proven to result in considerably different radiation patterns. From the seismotectonic point of view, the difference in fault plane solutions can be explained by an active growth of the seismogenic Ravne fault at its NW end, close to the structural barrier of the Bovec basin. Presumably, the fault is here split at the surface in two branches (Figure 1), but its behaviour at seismogenic depths is not known.

Comparison of the radiation patterns in the three orthogonal directions has shown that the SH component, which represents mainly Love waves, is the most important to understand the variations between macroseismic fields. For both earthquakes, the highest intensities were systematically observed in directions of four (1998) or two (2004) large amplitude lobes in SH component, which have significantly different orientation (for approx. 30°) for both events. This is in agreement with the fact that the most powerful vibrations from an earthquake at near distances are related to S-waves and Love waves [22]. No particular correlation was found between distribution of intensities and SV direction of the radiation pattern, which is characterized by medium amplitudes and almost symmetrical lobes. On the other hand, radial P direction, which is almost purely symmetrical for the strike-slip mechanism of the 1998 event, showed an interesting feature for the 2004 event. Its small reverse, steep dipping component of movement on predominantly strike-slip fault, has resulted in a very pronounced amplitude lobe in SW direction, where two settlements are located that express higher intensity in the 2004 event.

Macroseismic data inherit a certain degree of uncertainty due to lack of data in some settlements and due to subjectivity in intensity assessment, which was in our case made even more complex due to the following three factors. The first factor is caused by multiple damaging earthquakes in the same epicentral area. The assessment of intensity of the second event can be biased due to the damage from the first one, especially where the retrofitting was not yet finished. The second factor is related to the prominent site effects observed in the area and thus large variations in intensity at similar epicentral distances. The third factor is related to the fact that epicentral area is very sparsely populated due to high mountains environment. The macroseismic field is thus very unevenly sampled. Analyses of the influence of radiation pattern on it would be much easier, if the data at all azimuths and epicentral distances would be available. Nevertheless, by detailed analyses of fault mechanism radiation patterns we were able to explain some unusual differences between the observed macroseismic fields of 1998 and 2004 Krn Mountains earthquakes.

## Figures and Tables

**Table 1:** Fault plane solutions of 1998 and 2004 earthquakes in Krn Mountains.

Date	Time	Mw	Strike	Dip	Rake	Reference
12.4.1998	10:55	5.6	132	86	178	[1]
12.7.2004	13:04	5.2	99	72	156	[19]

**Table 2:** Intensities (EMS-98) of 1998 and 2004 earthquakes in some settlements in the Upper Soča valley and their difference (2004–1998).

Settlement	1998 intensity	2004 intensity	Intensity difference
Bovec	VII	VI-VII	−0.5
Čezsoča	VI-VII	VI-VII	0
Kal-Koritnica	VII	VI	−1
Vodenca	No data	VI-VII	
Lepena	VII-VIII	VI	−1.5
Soča	VI-VII	VI	−0.5
Magozd	VII-VIII	VI	−1.5
Sp. Drežniške Ravne	VII-VIII	V-VI	−2
Zg. Drežniške Ravne	VII	V-VI	−1.5
Drežnica	VI-VII	VI	−0.5
Kobarid	VI-VII	VI	−0.5
Tolminske Ravne	VII-VIII	No data	
Žaga	V-VI	V-VI	0
Srpenica	V-VI	VI	+0.5
Trnovo ob Soči	V-VI	VI	+0.5
Vrsno	VI	V-VI	−0.5

## References

[1] Zupančič P, Cecić I, Gosar A, Placer L, Poljak M, Živčić M (2001). The earthquake of 12 April 1998 in the Krn Mountains (Upper Soča valley, Slovenia) and its seismotectonic characteristics. *Geologija*.

[2] Cecić I, Živčić M, Jesenko T, Kolar J (2006). Potresi v Sloveniji leta 2004. Earthquakes in year 2004. *Slovenian Environment Agency*.

[3] Gosar A, Stopar R, Car M, Mucciarelli M (2001). The earthquake on 12 April 1998 in the Krn mountains (Slovenia): ground-motion amplification study using microtremors and modelling based on geophysical data. *Journal of Applied Geophysics*.

[4] Gosar A (2007). Microtremor HVSR study for assessing site effects in the Bovec basin (NW Slovenia) related to 1998 Mw5.6 and 2004 Mw5.2 earthquakes. *Engineering Geology*.

[5] Gosar A (2008). Site effects study in a shallow glaciofluvial basin using H/V spectral ratios from ambient noise and earthquake data: the case of bovec basin (NW Slovenia). *Journal of Earthquake Engineering*.

[6] Gosar A (2010). Site effects and soil-structure resonance study in the Kobarid basin (NW Slovenia) using microtremors. *Natural Hazards and Earth System Sciences*.

[7] Vidrih R, Ribičič M (2004). Potres 12. julija 2004 v zgornjem Posočju—preliminarne geološke in seizmološke značilnosti. *Geologija*.

[8] Sirovich L, Pettenati F (2009). Validation of a kinematic and parametric approach to calculating intensity scenarios. *Soil Dynamics and Earthquake Engineering*.

[9] Sirovich L, Pettenati F, Sandron D (2009). Source- and site-effects in the intensities of the M 5.4 29 july 2008 earthquake in South Los Angeles. *Seismological Research Letters*.

[10] Perniola B, Bressan G, Pondrelli S (2004). Changes in failure stress and stress transfer during the 1976-77 Friuli earthquake sequence. *Geophysical Journal International*.

[11] Kastelic V, Vrabec M, Cunningham D, Gosar A (2008). Neo-Alpine structural evolution and present-day tectonic activity of the eastern Southern Alps: the case of the Ravne Fault, NW Slovenia. *Journal of Structural Geology*.

[12] Aki K, Richards PG (2002). *Quantitative Seismology*.

[13] Bormann P, Wendt S, Starke U, Bormann P (2012). Radiation patterns of earthquake fault mechanisms. *New Manual of Seismological Observatory Practice*.

[14] Fitzko F, Suhadolc P, Aoudia A, Panza GF (2005). Constraints on the location and mechanism of the 1511 Western-Slovenia earthquake from active tectonics and modeling of macroseismic data. *Tectonophysics*.

[15] Camassi R, Caracciolo CH, Castelli V, Slejko D (2011). The 1511 Eastern Alps earthquakes: a critical update and comparison of existing macroseismic datasets. *Journal of Seismology*.

[16] Lapajne J, Šket-Motnikar B, Zupančič P (2001). Design ground acceleration map of Slovenia. Earthquakes in year 1999. *Slovenian Environment Agency*.

[17] Cunningham D, Grebby S, Tansey K, Gosar A, Kastelic V (2006). Application of airborne LiDAR to mapping seismogenic faults in forested mountainous terrain, southeastern Alps, Slovenia. *Geophysical Research Letters*.

[18] Bajc J, Aoudia A, Saraò A, Suhadolc P (2001). The 1998 Bovec-Krn mountain (Slovenia) earthquake sequence. *Geophysical Research Letters*.

[19] Kastelic V (2008). *Seismotectonic study of Ravne Fault and 1998 and 2004 Upper Posočje Earthquakes [Ph.D. thesis]*.

[20] Grünthal G (1998). *European Macroseimic Scale 1998*.

[21] Gosar A (2012). Application of Environmental Seismic Intensity scale (ESI, 2007) to Krn Mountains 1998 Mw = 5.6 earthquake (NW Slovenia) with emphasis on rockfalls. *Natural Hazards and Earth System Sciences*.

[22] Boatwright J, Choy GL (1992). Acceleration source spectra anticipated for large earthquakes in northeastern North America. *Bulletin of the Seismological Society of America*.

